# Short-term effects of COVID-19 pandemic on academic achievement among Colombian adolescents

**DOI:** 10.15446/rsap.V25n2.106463

**Published:** 2023-03-01

**Authors:** Jose Moreno-Montoya, Álvaro J. Idrovo, Silvia M. Ballesteros

**Affiliations:** 1 JM: Statis. M. Sc. Clinical Epidemiology. Ph.D. Epidemiology Sciences. Public Health Department, School of Medicine, Universidad Industrial de Santander. Bucaramanga, Colombia. jomormuq@uis.edu.co Universidad Industrial de Santander Public Health Department School of Medicine Universidad Industrial de Santander Bucaramanga Colombia jomormuq@uis.edu.co; 2 AI: Physcian. M. Sc. Public Health. Ph.D. Epidemiology Sciences. Public Health Department, School of Medicine, Universidad Industrial de Santander. Bucaramanga, Colombia. idrovoaj@uis.edu.co Universidad Industrial de Santander Public Health Department School of Medicine Universidad Industrial de Santander Bucaramanga Colombia idrovoaj@uis.edu.co; 3 SB: Physiotherapist. M Sc. Epidemiology. Clinical Studies and Epidemiology Division, Fundación Santa Fe de Bogotá. Bogotá, Colombia. smballesterosr@gmail.com Epidemiology Clinical Studies and Epidemiology Division Fundación Santa Fe de Bogotá Bogotá Colombia

**Keywords:** Educational achievement, health inequities, socioeconomic Status, COVID-19, ethnicity, child labour *(source: MeSH, NLM)*, Logros educativos, inequidades en salud, nivel socioeconómico, COVID-19, etnicidad, trabajo infantil *(fuente: DeCS, BIREME)*

## Abstract

**Objective:**

The aim of this study was to assess the effect of health-related determinants and COVID-19 pandemic on the academic achievement of Colombian youth.

**Methods:**

Nationwide study based on the results of official exams of more than two million students during the period 2017-2020. Sociodemographic characteristics, dietary, ethnicity, child labour factor, and region-level rurality were considered as independent variables. A two-level structural equation model was used to assess the effect of individual- and state-level variables. Analyses were stratified by academic domains and global score.

**Results:**

Health-related determinants, including belonging to an ethnic minority and child labour were associated with a reduction in global scores (20.07, 95 % CI 19.81-20.33 and 10.62, 95 % CI 10.49-10.76 points, respectively), whereas the youth from higher socioeconomic status achieved a 2.21 points increase. COVID-19 pandemic and rurality did not implied significant changes in the scores, however, rurality was associated with a reduction of 0.01 points in foreign language score (English).

**Conclusions:**

Health determinants not only affect the common outcomes in health but also explain educational inequalities in Colombian youth. Beyond an increased risk of morbidity or mortality, as reported elsewhere, belonging to a minority, coming from a lower socioeconomic stratum and be in need to work, put in risk the personal fulfilment of youth, which entail poor future health performance. A more comprehensive analysis of health determinants and its consequences is needed in young people.

## RESUMEN

**Objetivo:**

El objetivo de este estudio fue evaluar el efecto de los determinantes relacionados con la salud y la pandemia de COVID-19 en el rendimiento académico de los jóvenes colombianos.

**Métodos:**

Estudio a nivel nacional basado en los resultados de exámenes oficiales de más de dos millones de estudiantes durante el período 2017-2020. Las características sociodemográficas, la dieta, el origen étnico, el factor de trabajo infantil y la ruralidad a nivel regional se consideraron variables independientes. Se utilizó un modelo de ecuaciones estructurales de dos niveles para evaluar el efecto de las variables a nivel individual y estatal. Los análisis se estratificaron por dominios académicos y puntuación global.

**Resultados:**

Los determinantes relacionados con la salud, incluida la pertenencia a una minoría étnica y el trabajo infantil, se asociaron con una reducción en las puntuaciones globales (20,07, IC 95 % 19,81-20,33 y 10,62, IC 95 % 10,49-10,76 puntos, respectivamente), mientras que los jóvenes de mayor nivel socioeconómico lograron un aumento de 2,21 puntos. La pandemia de COVID-19 y la ruralidad no implicaron cambios significativos en los puntajes, sin embargo, la ruralidad se asoció con una reducción de 0,01 puntos en el puntaje de lengua extranjera (inglés).

**Conclusiones:**

Los determinantes de la salud no solo afectan a los resultados comunes en salud, sino que explican las desigualdades educativas en la juventud colombiana. Más allá de un mayor riesgo de morbilidad o mortalidad, como se informó en otros lugares, pertenecer a una minoría, provenir de un estrato socioeconómico más bajo y estar en necesidad de trabajar, pone en riesgo la realización personal de los jóvenes, lo que implica un bajo desempeño futuro en salud. Se necesita un análisis más exhaustivo de los determinantes de la salud y sus consecuencias en los jóvenes.

The COVID-19 pandemic has caused an increase in infections, illnesses, suffering, hospitalizations and deaths around the world. Moreover, there are non-health effects that can have enormous impacts on societies, such as food and housing insecurity, decreased family income and social status, increased abuse, mistreatment, racial or ethnic discrimination, and educational disruption 1,2. The deterioration of education is specially relevant, since its consequences may be related with short- and long-term social and health adverse outcomes 3, setting up a vicious circle. One of the non-pharmacological measures to manage the pandemic was confinement, including the closure of schools 4. Although they were useful to reduce the transmission of COVID-19, some estimates from the World Health Organization suggest that the negative effects depend on the duration of school closure 2, thus, the situation was assessed as a global catastrophe 5 in countries like Colombia.

In this country, some studies prior to the pandemic reported that the lowest academic achievement scores are observed among the most vulnerable students 6,7. In addition, Colombia was one of the countries with the longest duration of school closures (from March 2020 to September 2021 - January 2022, depending on each institution) 8, therefore, adverse effects associated with lockdown and school closures may occur. The closure and reopening of educational institutions was deter-mined by the national government 8, however, in many cases parents did not allow their children to return to face-to-face classes 9.

In this study, we explored the potential associations between some social determinants and academic achievements among Colombian adolescents. We included national data from 2017 to 2020 to identify changes between pre-pandemic and pandemic times. Only short-term effects were analyzed among adolescents who finished high school. Although this population group does not usually have severe COVID-19 10, it is possible that they have adverse effects on their mental health 11,12, with repercussions on their school performance.

## MATERIALS AND METHODS

Settings and participants. Official results of national academic tests between 2017 and 2020 were analysed. The tests are applied by the Colombian Institute for the Evaluation of Education and are aimed to evaluate the skills and academic achievement of Colombian secondary school students in conjunction with their background, non-cognitive characteristics and school context 13. The exam is applied since 1968 every six months and is required for college and university admission; it includes questions regarding familiar socioeconomic traits such as household belongings and parents' education level; students' ethnicity, working status and food frequency intake. The academic achievement is assessed through the domains of Critical Reading, Mathematics, Social and Citizenship, Natural Sciences and English as foreign language; a global score which is the weighted average of the five scores is calculated as follows: Global Score = 5 * ((3^*^Math + 3*Reading + 3^*^Natural science + 3^*^Social + 1^*^English) / 13) 13.

Variables. Scores of each domain and the global score were considered as the dependent variables. Scores varied from 0 and 100 for the five domains, and from 0 to 500 for the global score 13. Independent individual-level variables were ethnicity, defined as belonging or not to an ethnic minority; adolescent labour, defined as the self-report of working outside the house and school the previous week of the exam; food frequency intake per week of dairy products, meat and eggs, and cereals, nuts or legumes through a Likert scale, going from never or almost never to daily or almost daily; and socioeconomic status (SES) by means of a composite indicator that includes the educational level of parents, material of floors and walls of the house, toilets and proper sanitation facilities, internet and television services, and other amenities including computer, DVD, refrigerator and washing machine 14. This indicator ranges between 0 and 100, being 0 the less favoured. At collective level, Colombian states were considered as natural grouping variables, and rurality was included in the model by means of the proportion of people living in rural places, i.e., the total of people living in rurality divided by the total population of the state by year 15.

Statistical analysis. Global and specific scores for each academic domain were reported using means and standard deviations according to sociodemographic characteristics. A crude evaluation of the effect of individual-level variables was obtained using Wald tests, and associations between rurality and academic domains were assessed using Pearson correlation coefficients. Two-level structural equation models (SEM) were used to evaluate the effect of individual-level variables and rurality on academic scores. Covariance among individual variables and rurality were considered in the model. P-values less than 0.05 were considered significant. All analyses were carried out via Stata V.17 (Stata, Inc., College Station, TX, US) 16.

During the study period, a total of 2,147,062 adolescents were included distributed as follows: 546,255 (25.4 %; 95 % CI 25.4 %-2 5.5%) in 2017, 549,933 (25.6 %; 25.6 %o-2 5.7 %) in 2018, 546,077 (25.4 %; 25.4 %o-2 5.5 %) in 2019, and 504,797 (23.5 %; 23.5 %o-23.6 %) in 2020 (Table 2). 54.4 % (95 % CI 54.3 %-54.5 %) of the people included in this study were females, 6.6 % (6.5 %-6.6 %) belonged to an ethnic minority, 38.6 % (38.5 %-38.6 %) reported not having access to internet and 34.6 % (34.5 90-34.7 %) worked the previous week to the exam. The SES score varied from 12.5 to 84.8 (mean=50.3, SD=9.5), and less than the 50 % of students reported a daily or almost daily intake of meat or eggs (39.9 %; 39.9 %>-40.0 %), dairy products (30.6 %; 30.5 %o-30.6 %), and cereal, nuts, or legumes (16.6 %; 16.5 %-16.6%). Table 1.

**Table 1:** Individual characteristics and academic scores

	Critical reading			Mathematics		Citizen social science		Natural sciences		English		Global
	Mean (SD)	p	Mean (SD)	p	Mean (SD)	p	Mean (SD)	p	Mean (SD)	p	Mean (SD)	p
Year												
2017 (n = 546 255, 24.4 %)	53.19 (9.80)		50.06 (12.02)		50.23 (11.12)		51.22 (10.04)		49.52 (11.46)		255.23 (48.48)	
2018 (n = 549 933, 25.6 %)	52.56 (10.13)	<0.001	50.19 (11.95)	<0.001	48.19 (11.78)	<0.001	49.53 (10.45)	<0.001	50.60 (11.53)	<0.001	250.78 (50.09)	<0.001
2019 (n = 546 077, 25.4 %)	52.16 (10.54)		50.61 (11.99)		46.23 (12.14)		48.24 (10.76)		48.42 (12.56)		246.19 (51.39)	
2020 (n = 504 797, 23.5 %)	52.16 (10.16)		51.02 (11.65)		48.23 (11.97)		48.20 (10.50)		46.88 (11.31)		248.35 (48.69)	
Ethnic group												
Yes (n = 2 001 514, 93.4 %)	46.60 (0.03)	<0.001	43.71 (0.03)	<0.001	41.95 (0.03)	<0.001	43.58 (0.03)	<0.001	42.98 (0.03)	<0.001	219.41 (0.12)	<0.001
No (n = 141 700, 6.6 %)	52.95 (0.01)		50.94 (0.01)		48.67 (0.01)		49.73 (0.01)		49.32 (0.01)		252.37 (0.03)	
Child labour												
Yes (n = 721 546, 34.6 %)	49.75 (0.01)	<0.001	47.76 (0.01)	<0.001	45.21 (0.01)	<0.001	46.63 (0.01)	<0.001	45.43 (0.01)	<0.001	235.95 (0.05)	<0.001
No (n = 1 363 893, 65.4 %)	54.09 (0.01)		52.03 (0.01)		49.93 (0.01)		50.91 (0.01)		50.89 (0.01)		258.38 (0.04)	
Internet access												
Yes (n = 1 254 248, 61.4 %)	54.78 (0.01)	<0.001	53.08 (0.01)	<0.001	50.73 (0.01)	<0.001	51.51 (0.01)	<0.001	51.94 (0.01)	<0.001	262.40 (0.04)	<0.001
No (n = 787 133, 38.6 %)	49.43 (0.01)		47.03 (0.01)		44.82 (0.01)		46.61 (0.01)		44.72 (0.01)		233.99 (0.05)	
Dairy intake												
Never/almost never (n = 186 032, 9.2 %)	48.46 (0.02)		45.60 (0.03)		43.80 (0.02)		45.35 (0.02)		44.37 (0.02)		228.46 (0.10)	
1 or 2 times per week (n = 696 459, 34.3 %)	50.80 (0.01)	<0.001	48.48 (0.01)	<0.001	46.22 (0.01)	<0.001	47.67 (0.01)	<0.001	46.49 (0.01)	<0.001	240.77 (0.05)	<0.001
3 to 5 times per week (n = 527 277, 26.0 %)	53.63 (0.01)		51.89 (0.02)		49.37 (0.02)		50.42 (0.01)		49.78 (0.02)		256.05 (0.07)	
Every/almost every day (n = 620 711, 30.6 %)	55.36 (0.01)		53.85 (0.02)		51.57 (0.02)		52.43 (0.01)		53.07 (0.02)		266.43 (0.07)	
Meat, fish or eggs intake												
Never/almost never (n = 97 265, 4.8 %)	48.41 (0.03)		45.26 (0.04)		43.86 (0.04)		44.88 (0.03)		44.32 (0.03)		227.53 (0.15)	
1 or 2 times per week (n = 505 946, 24.9 %)	50.33 (0.01)	<0.001	47.83 (0.02)	<0.001	45.81 (0.02)	<0.001	47.12 (0.01)	<0.001	45.99 (0.01)	<0.001	238.18 (0.06)	<0.001
3 to 5 times per week (n = 619 351, 30.4 %)	52.84 (0.01)		50.91 (0.01)		48.48 (0.01)		49.65 (0.01)		48.83 (0.01)		251.72 (0.06)	
Every/almost every day (n = 812 559, 39.9 %)	54.61 (0.01)		53.07 (0.01)		50.60 (0.01)		51.72 (0.01)		51.95 (0.01)		262.28 (0.06)	
Cereals, nuts or legumes intake												
Never/almost never (n = 292 533, 14.4 %)	50.12 (0.02)		47.60 (0.02)		45.45 (0.02)		46.93 (0.02)		45.89 (0.02)		236.98 (0.09)	
1 or 2 times per week (n = 796 912, 39.3 %)	51.92 (0.01)	<0.001	49.95 (0.01)	<0.001	47.48 (0.01)	<0.001	48.87 (0.01)	<0.001	48.04 (0.01)	<0.001	247.20 (0.05)	<0.001
3 to 5 times per week (n = 603 120, 29.7 %)	54.21 (0.01)		52.57 (0.02)		50.18 (0.02)		51.15 (0.01)		50.85 (0.02)		259.68 (0.06)	
Every/almost every day (n = 335 704, 16.6 %)	54.17 (0.02)		52.07 (0.02)		50.26 (0.02)		51 .04 (0.02)		51.68 (0.02)		259.34 (0.09)	
Socioeconomic status (mean=50.3; SD=9.5) Correlation coefficient	0.389		0.388		0.379		0.382		0.486		0.440	

**Table 2:** Correlations between rural population proportion and academic scores

	Critical reading	Math	Natural science	Citizen social	English	Global
Rural population %	-0.135	-0.111	-0.107	-0.122	-0.162	-0.137
Critical reading	1.000	0.729	0.740	0.794	0.654	0.894
Math		1.000	0.778	0.723	0.647	0.894
Natural science			1.000	0.784	0.689	0.910
Citizen social				1.000	0.664	0.912
English					1.000	0.774

A total of 580,717 persons resided in the 4 biggest cities in the country: Bogotá, Medellin, Barranquilla and Cali; 338,459 (15.8 %>; 95 %o CI 15.7 %>-15.8 %o) were from Bogotá, the capital city. In contrast, a 15.6 % (95 % CI 15.6 %-15.7 %; N=334,997) of the scholars studied in institutes located in rural areas.

Average academic scores by domain varied from 48.22 (SD = 11.84; 95 % CI 48.20-48.24) in citizen social science to 52.52 (SD=10.17; 95 % CI 52.51-52.54) in critical reading. The highest average scores, both global and specific domains, were reported in Bogotá, also the area with the lowest proportion of rural residents (0.2 %). Correlation between rurality and average scores are shown in Table 2.

Crude comparisons showed significant effects of ethnic minority, child labour, and low food frequency intake on each of the five domains and the global score (Table 1). In the adjusted analyses, individual-level variables remained significant; in particular, belonging to an ethnic minority reduced the scores on average by 2.83 (95 % CI 2.77-2.89) and 4.61 (4.55- 4.67) in English and Mathematics tests, respectively. Likewise, global score was 20.07 (19.8120.33) points less among minority students. Adolescent labour was also associated with lower test results, with 1.72 (1.69-1.75) and 2.35 (2.32-2.38) points less in mathematics and social sciences, respectively; and an average reduction of 10.62 (10.49-10.76) points in the global score. Regarding the SES punctuation, an average increase of 2.21 (2.20-2.22) points in the global results is shown with an increase of one point in the SES indicator. Food intake effects varied from a reduction of 0.06 (0.04-0.07) points in social science scores with higher consumption of cereals and legumes to an increase of 1.29 (1.22-1.37) in global score with higher dairy consumption. Dairy intake did not had a significant effect on English test results (p=0.068). Rurality was positively associated with all academic domains, except for English language. As expected, individual level variables showed a significant correlation (Table 3).

**Table 3:** Adjusted effects of individual characteristics and rural residence on academic scores. Structural equation modelling

	Mathematics			Critical reading			Natural sciences			Social sciences			English language			Global score		
Direct effects	Coeff.	95 %IC		Coeff.	95 %IC		Coeff.	95 %IC		Coeff.	95 %IC		Coeff.	95 %IC		Coeff.	95 %IC	
Individual-level variables																		
Internet access	-0.59	-0.63	-0.55	-0.24	-0.28	-0.21	-1.09	-1.12	-1.05	-0.59	-0.63	-0.55	-1.49	-1.53	-1.45	-3.48	-3.65	-3.30
SES	0.47	0.46	0.47	0.38	0.37	0.38	0.41	0.41	0.42	0.45	0.44	0.45	0.63	0.63	0.64	2.21	2.20	2.22
Child labour	-1.72	-1.75	-1.69	-2.25	-2.28	-2.22	-2.11	-2.12	-2.08	-2.35	-2.38	-2.32	-2.34	-2.37	-2.31	-10.62	-10.76	-10.49
Dairy intake	0.33	0.31	0.35	0.24	0.23	0.26	0.25	0.24	0.27	0.30	0.28	0.32	-0.02*	-0.03	0.00	1.29	1.22	1.37
Meat intakea	0.08	0.06	0.10	-0.10	-0.12	-0.09	0.08	0.06	0.10	-0.23	-0.24	-0.21	-0.38	-0.40	-0.36	-0.35	-0.43	-0.27
Legumes intakeb	-0.33	-0.35	-0.31	-0.12	-0.13	-0.10	-0.20	-0.22	-0.19	-0.06	-0.07	-0.04	-0.25	-0.26	-0.23	-0.91	-0.98	-0.83
Minority	-4.61	-4.67	-4.55	-3.89	-3.94	-3.83	-3.92	-3.98	-3.87	-4.02	-4.09	-3.96	-2.83	-2.89	-2.77	-20.07	-20.33	-19.81
State-level variable																		
Rurality	0.02	0.02	0.02	0.00	0.00	0.00	0.02	0.02	0.02	0.01	0.01	0.01	-0.01	-0.01	-0.01	0.06	0.05	0.06
Non-direct effects																		
SES -> Internet access	0.03	0.03	0.03	0.03	0.03	0.03	0.03	0.03	0.03	0.03	0.03	0.03	0.03	0.03	0.03	0.03	0.03	0.03
Internet access -> Year of exam	0.21	0.22	0.21	0.22	0.22	0.21	0.22	0.22	0.21	0.22	0.22	0.21	0.22	0.22	0.21	0.22	0.22	0.21
SES -> Worked previous week	-0.01	-0.01	-0.01	-0.01	-0.01	-0.01	-0.01	-0.01	-0.01	-0.01	-0.01	-0.01	-0.01	-0.01	-0.01	-0.01	-0.01	-0.01
SES <-> Dairy intake	5.07	5.06	5.09	5.07	5.06	5.09	5.07	5.06	5.09	5.07	5.06	5.09	5.07	5.06	5.08	5.07	5.06	5.09
SES <-> Meat intakea	4.27	4.25	4.28	4.27	4.25	4.28	4.27	4.25	4.28	4.27	4.25	4.28	4.27	4.25	4.28	4.27	4.25	4.28
SES <-> Legumes intakeb	3.39	3.38	3.41	3.39	3.38	3.41	3.39	3.38	3.41	3.39	3.38	3.41	3.39	3.38	3.41	3.39	3.38	3.41
SES <-> State-level Rurality	-45.39	-45.61	-45.17	-45.39	-45.61	-45.17	-45.39	-45.61	-45.17	-45.39	-45.61	-45.17	-45.37	-45.58	-45.15	-45.39	-45.61	-45.17
SES <-> Minority ethnic group	-0.36	-0.36	-0.36	-0.36	-0.36	-0.36	-0.36	-0.36	-0.36	-0.36	-0.36	-0.36	-0.36	-0.36	-0.36	-0.36	-0.36	-0.36
Dairy intake <-> Meat intakea	0.39	0.39	0.39	0.39	0.39	0.39	0.39	0.39	0.39	0.39	0.39	0.39	0.39	0.39	0.39	0.39	0.39	0.39
Dairy intake <-> Legumes intakeb	0.32	0.32	0.32	0.32	0.32	0.32	0.32	0.32	0.32	0.32	0.32	0.32	0.32	0.32	0.32	0.32	0.32	0.32
Dairy intake <-> State-level Rurality	-3.09	-3.11	-3.07	-3.09	-3.11	-3.07	-3.09	-3.11	-3.07	-3.09	-3.11	-3.07	-3.09	-3.11	-3.07	-3.09	-3.11	-3.07
Dairy intake <-> Minority ethnic group	-0.03	-0.03	-0.03	-0.03	-0.03	-0.03	-0.03	-0.03	-0.03	-0.03	-0.03	-0.03	-0.03	-0.03	-0.03	-0.03	-0.03	-0.03
Meat intakea <-> Legumes intakeb	0.27	0.27	0.27	0.27	0.27	0.27	0.27	0.27	0.27	0.27	0.27	0.27	0.27	0.27	0.27	0.27	0.27	0.27
Meat intakea <-> State-level Rurality	-2.53	-2.55	-2.51	-2.53	-2.55	-2.51	-2.53	-2.55	-2.51	-2.53	-2.55	-2.51	-2.53	-2.55	-2.51	-2.53	-2.55	-2.51
Meat intakea <-> Minority ethnic group	-0.02	-0.02	-0.02	-0.02	-0.02	-0.02	-0.02	-0.02	-0.02	-0.02	-0.02	-0.02	-0.02	-0.02	-0.02	-0.02	-0.02	-0.02
Legumes intakeb <-> State-level Rurality	-1.38	-1.40	-1.36	-1.38	-1.40	-1.36	-1.38	-1.40	-1.36	-1.38	-1.40	-1.36	-1.38	-1.40	-1.36	-1.38	-1.40	-1.36
Legumes intakeb <-> Minority ethnic group	-0.02	-0.02	-0.02	-0.02	-0.02	-0.02	-0.02	-0.02	-0.02	-0.02	-0.02	-0.02	-0.02	-0.02	-0.01	-0.02	-0.02	-0.02
State-level Rurality <-> Minority ethnic group	0.94	0.94	0.95	0.94	0.94	0.95	0.94	0.94	0.95	0.94	0.94	0.95	0.94	0.94	0.95	0.94	0.94	0.95

^a^Meat, fish or eggs intake; bCereals, nuts or legumes intake; ^*^p<0.05 All other estimates were p<0.001.

## DISCUSSION

In this study, based on official records of the academic state exams from 2017 to 2020, we verified that recognized social determinants have also a significant negative effect in the academic achievement in Colombian youth, and in consequence, a poor performance in near future regarding personal fulfilment is expected. However, no differences in academic achievement were identified between pre-pandemic pandemic times. Similar to previous health studies in the region, variables like food insecurity, poor access to health services and poverty 17, are simultaneously related to increased morbidity, mortality, poor quality of life and decreased academic attainment. Indeed, our results indicated that a higher socioeconomic level confers an increase of approximately 2 points in the global test score with specific increases in each domain once compared with people living in less favoured conditions. Comparable findings have also been showed about socioeconomic status and mortality and morbidity elsewhere 18.

Previous adolescent labour was also identified as a negative predictor of academic accomplishment. In this study, the participants who reported having worked "during the last week", had worse scores in both the global and specific scores. A reduction over 10 points in the final rating was evidenced. As reported before, adolescents who have to work usually observe many other risk factors for their development, including poverty, mistreatment, violence and malnutrition 19. Social and family poverty, loss or incapacitation/illness of parents, lack of social security and protection, and ignorance about the value of, or limited access to, education are among the myriad reasons for the involvement of adolescents in the workforce, but all of them are simultaneously involved in a network of factors that at the same time affect the health and development opportunities of youths 20.

Malnutrition during childhood is also a wide discussed health factor and its short- and long-term implications seem to be clear 21. Consumption of animal source foods in children has been associated with a reduced likelihood of stunting 22. In our context, as expected, malnutrition appears to be associated with lower competitive academic achievement as well as having lower self-esteem during adulthood 23. Our results showed an important reduction in every score analysed among youth who eat meet or dairy products less frequently.

The biggest reduction, however, was associated to those who belong to a minority ethnic group. About 20 points less are expected in those who self-reported belonging to an ethnicity. Long-term barriers to health and well-being have been previously identified everywhere regarding minorities 24, and this finding endorses the fact that this population faces not only difficulties related to their health and social exclusion, but also long-term disadvantages due to reduced academic options and fewer opportunities to reach college education, which, as is known, shall redound in poor health and life quality. Deep health inequities are expectable in minority ethnic groups all over the world, most severe in developing countries, and with several negative consequences, including, but not limited to, unequal access to screening and health services, poor working conditions, higher levels of forced migration and violence 25, all of which are profoundly aggravated by educational limitations.

About rurality, our results showed only slight differences (0.01 to 0.06 points) in the scores, but a negative correlation exists among rurality proportion of population and the global score and specific for every domain. Likewise, rural areas scores are probably affected due that the figures only take into account the students that did not dropout school. Dropout rates have reported to be higher in rural contexts 26.

Some limitations need to be declared to put in context the findings. First, the developed analyses supposes that state exams measure all Colombian youth, but the records are not relevant figures on people who did not finish secondary education. In the same way, a reliable level of population coverage of the exams is not offered in the official records. In the country, for 2019, the school dropout rate of the official sector was of 3.2 % 27. Low academic performance has been described as one of the main predictors of school dropout 28. Second, measures including student's age and about the institutions facilities, infrastructure, violence conditions, number or teachers per student, and many others at institution level are not available for this analysis and could affect our conclusions, nonetheless, we considered that these variables are indirectly related to the results. According to previous authors, disadvantaged and minority children are much more concentrated in highly poor schools 29.

However, our results demonstrate the importance of widely recognized social determinants in explaining educational inequalities, and of the local educational context in determining the possibility of educational attainment of students from lower socioeconomic status or with minority backgrounds. Actions to manage those inequities, both within schools and in wider society, are urgently required to help address these educational disparities, which, in turn, may affect the adolescents in their own present health and their subsequent health in adult life. Despite the fact that pandemic had no effects on academic achievement among the youth in this study, some containment strategies, such as lockdowns and school closures, exacerbated health, social, economic, and academic inequalities 30, therefore, it is possible that in younger children the effects will be seen in the future. It is also plausible that the effects among adolescents would be observed afterwards, in technical, technological or university studies, as well as in work as adults. Further studies with these student cohorts will be essential to understand the non-health problems of the pandemic, and define the appropriate actions to mitigate the effect ♠

## References

[1] Abrams EM, Greenhawt M, Shaker M, Pinto AD, Sinha I, Singer A (2022). The COVID-19 pandemic: Adverse effects on the social determinants of health in children and families. Ann Allergy Asthma Immunol.

[2] Azevedo JP, Hasan A, Goldemberg D, Geven K, Iqbal SA (2021). Simulating the potential impacts of COVID-19 school closures on schooling and learning outcomes: a set of global estimates. World Bank Res Obs.

[3] Reimers FM, Reimers FM (2022). Primary and secondary education during COVID-19: disruptions to educational opportunity during a pandemic.

[4] Hale T, Angrist N, Goldszmidt R, Kira B, Petherick A, Phillips T (2021). A global panel database of pandemic policies (Oxford COVID-19 Government Response Tracker). Nat Hum Behav.

[5] Buonsenso D, Roland D, De Rose C, Vásquez-Hoyos P, Ramly B, Chakakala-Chaziya JN (2021). Schools closures during the COVID-19 pandemic: a catastrophic global situation. Pediatr Infect Dis J.

[6] Galvis-Molano DL, Silva-Arias AC, Sarmiento-Espinel JA (2022). Logro educativo de las instituciones con estudiantes de minorias étnicas en Colombia. Entramado.

[7] Gómez Soler SC (2017). Civil conflict and educational achievement: the case of the Colombian secondary school exit examination. Colombia Int.

[8] Knaul FM, Touchton MM, Arreola-Ornelas H, Calderon-Anyosa R, Otero-Bahamón S, Hummel C (2022). Strengthening health systems to face pandemics: subnational policy responses to COVID-19 in Latin America. Health Aff (Millwood).

[9] Alvarado-Socarrás J, Quintero-Lesmes DC, Carmona JC, Niederbacher-Velásquez J, Franco-López M (2021). Factores asociados a la decisión paterna sobre el retorno a clases presenciales en Colombia durante la pandemia COVID-19. Rev Univ Ind Santander Salud.

[10] Oliveira MCL, Colosimo EA, Vasconcelos MA, Martelli-Júnior H, Mak RH, Silva LR (2023). The association between pre-existing asthma and reduced risk of death among children and adolescents hospitalized with COVID-19 in Brazil. Pediatr Pulmonol.

[11] Taskesen B, Kardas O, Yilmaz K (2023). Evaluation of depression, anxiety and posttraumatic stress response levels of children and adolescents treated with COVID-19. Eur J Pediatr.

[12] Hussong AM, Benner AD, Erdem G, Lansford JE, Makila LM, Petrie RC (2021). Adolescence amid a pandemic: short- and long-term implications. J Res Adolesc.

[13] Colombian Institute for the Evaluation of Education (2017). Saber 11 Exam Documentation.

[14] Colombian Institute for the Evaluation of Education (2019). How is the Socioeconomic Level Index (INSE) constructed in the context of the Saber tests?.

[15] Agencia de Desarrollo Rural (2018). rural Statistical Bulletin.

[16] StataCorp (2019). Stata Statistical Software: Release 16.

[17] Hackett M, Melgar-Quiñonez H, Taylor CA, Alvarez Uribe MC (2010). Factors associated with household food security of participants of the MANA food supplement program in Colombia. Arch Latinoam Nutr.

[18] Signorello LB, Cohen SS, Williams DR, Munro HM, Hargreaves MK, Blot WJ (2014). Socioeconomic status, race, and mortality: a prospective cohort study. Am J Public Health.

[19] Ávila AS (2007). Trabajo infantil e inasistencia escolar. Rev Bras Educ.

[20] Kaur N, Byard RW (2021). Prevalence and potential consequences of child labour in India and the possible impact of COVID-19 - a contemporary overview. Med Sci Law.

[21] Khaliq A, Wraith D, Nambiar S, Miller Y (2022). A review of the prevalence, trends, and determinants of coexisting forms of malnutrition in neonates, infants, and children. BMC Public Health.

[22] Headey D, Hirvonen K, Hoddinott J (2018). Animal sourced foods and child stunting. Am J Agric Econ.

[23] Mwene-Batu P BG, Baguma M, Chabwine J, Bapolisi A, Chimanuka C, Molima C, Dramaix M, Kashama N, Macq J, Donnen P (2020). Long-term effects of severe acute malnutrition during childhood on adult cognitive, academic and behavioural development in African fragile countries: The Lwiro cohort study in Democratic Republic of the Congo. PLoS One.

[24] Sangaramoorthy M, Shariff-Marco S, Conroy SM, Yang J, Inamdar PP, Wu AH (2022). Joint associations of race, ethnicity, and socioeconomic status with mortality in the multiethnic cohort study.

[25] Sekhon Inderjit Singh HK, Lal N, Majeed A, Pawa N (2022). A systematic review of ethnic disparities in the uptake of colorectal cancer screening. Perspect Public Health.

[26] Gómez-Restrepo C, Muñoz AP, Rincón CJ (2016). Deserción escolar de adolescentes a partir de un estudio de corte transversal: Encuesta Nacional de Salud Mental Colombia 2015. Rev Colomb Psiquiatr.

[27] National Administrative Department of Statistics (2021). Technical Bulletin. Formal Education (EDUC) 2020.

[28] Wood L, Kiperman S, Esch RC, Leroux AJ (2017). Truscott SD. Predicting dropout using student- and school-level factors: An ecological perspective. Sch Psychol Q.

[29] Saporito S, Sohoni D (2007). Mapping educational inequality: concentrations of poverty among poor and minority students in public schools. Soc Forces.

[30] Goudeau S, Sanrey C, Stanczak A, Manstead A, Darnon C (2021). Why lock-down and distance learning during the COVID-19 pandemic are likely to increase the social class achievement gap. Nat Hum Behav.

